# Herniation of Small Bowel Loop through a Broad Ligament Defect Masquerading as Torsion of Ovarian Cyst

**DOI:** 10.1155/2013/246549

**Published:** 2013-12-26

**Authors:** Babatola Bakare, Olumide Akadiri, Akinyemi Akinsoji Akintayo

**Affiliations:** ^1^Department of Obstetrics and Gynaecology, Ondo State Specialist Hospital, Ondo State, Nigeria; ^2^Department of Obstetrics, Gynaecology, Ekiti State University Teaching Hospital, Ado Ekiti, Nigeria

## Abstract

Torsion of ovarian cyst is a common cause of acute abdomen especially in women of reproductive age-group. It commonly presents with colicky abdominal pain associated with nausea and vomiting. It could however mimic acute intestinal obstruction. The patient was a 32-year-old multipara with no previous history of pelvic or abdominal surgery. She was admitted with colicky lower abdominal pain associated with repeated episodes of vomiting and nausea. Laboratory investigations were essentially normal. Abdominopelvic USS showed a hypoechoic mass lesion in the left adnexium measuring 7.1 × 5.5 cm; surrounding bowel loops were hypoactive, dilated, and fluid filled. Diagnosis of acute abdomen secondary to suspected torsion of ovarian cyst was made. Management began for acute abdomen with intravenous hydration, prophylactic antibiotics, and analgesics. An emergency laparotomy revealed about 6 cm defect in the left broad ligament in which a 20 cm segment of terminal ileum was encased. Liberation of the ileal segment was done and the broad ligament defect closed. Bowel obstruction requires high index of suspicion in a patient with acute abdomen due to suspected torsion ovarian cyst most especially in the absence of previous pelvic or abdominal surgery.

## 1. Introduction 

Intestinal obstruction occurring from internal hernia is very rare, with a reported incidence between 0.2% and 0.8% [1, 2]. An internal hernia means herniation of hollow viscus usually the small intestine through a natural or an acquired opening within the peritoneal cavity [3].

An adnexal torsion accounts for roughly 3% of gynaecologic emergencies [4]. Adnexal torsion can occur in young girl [5] and is increasingly recognized as a cause of pelvic pain in postmenopausal women but is still most common in the reproductive years because of the regular development of a corpus luteal cyst during the menstrual cycles [6]. In adnexal torsion, venous and lymphatic obstruction occurs initially with subsequent congestion and edema of the ovary, which may progress to ischemia and necrosis and eventual infarction of the ovary [4].

**Figure 1:**
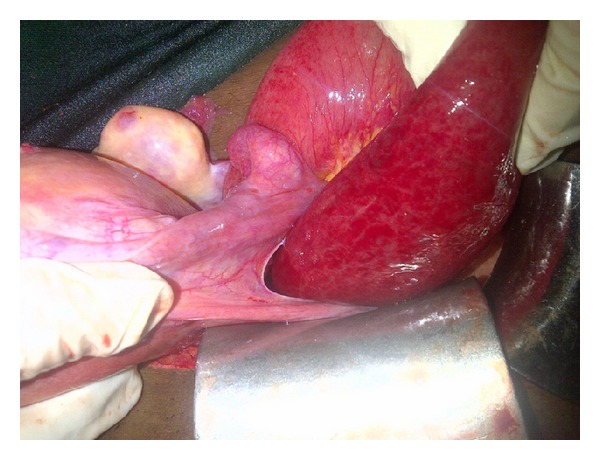
Herniated bowel through the defect in the broad ligament.

Adnexal torsion often can be a challenging diagnosis to make, because the classic symptoms of severe, sharp unilateral abdominal pain and nausea may not be present [7] and occasionally mimic exactly intestinal obstruction most especially in women with previous abdominal of pelvic surgery. The presence of known risk factors for adnexal torsion such as ovarian mass or infertility treatments may suggest the diagnosis [4]. Small bowel herniation through a defect in the broad ligament (Figure 1) could be a close differential of an adnexal torsion and clinical diagnosis before surgery may be extremely difficult. 

## 2. Case Presentation

This case concerns a 32-year-old P3+^0^ (3A), who presented complaining of acute lower abdomen colicky pain of 12-hour duration with nausea and repeated episodes of vomiting for 10 hours before presentation. Vomitus contained essentially recently ingested food initially but later bilious. She had no prior relevant medical history. There is no prior history of pelvic or abdominal surgery. On physical examination her left lower quadrant was tender with a palpable mass of about 6 cm by 4 cm, with mild voluntary guarding and increasing bowel sounds. Rectal examination did not show any abnormalities. Laboratory studies showed no definite abnormalities.

An urgent abdominopelvic ultrasound revealed a hypoechoic mass lesion in the left adnexia measuring 7.1 by 5.5 cm, with surrounding hypoactive, dilated and fluid filled. Supportive therapy was commenced with analgesics and prophylactic antibiotics which did not improve patient clinical condition. An urgent exploratory laparotomy was carried out which revealed a defect of about 6 cm in the left broad ligament between the left round ligament and the left fallopian tube through which about 20 cm length of the ileum had herniated (Figure 3). No evidence of vascular compromise was noticed change in the herniated bowel which was liberated from the defect and returned to the abdominal cavity. The defect on the broad ligament was also closed and the postoperative course was uneventful. She was discharged home on the fourth day after the operation (Figure 4).

## 3. Discussion

Internal hernia is responsible for about 0.9% of intestinal obstruction. Hernia of the broad ligament is extremely rare and accounted for less than 7% of all internal hernias.

The earliest reported case of an incarcerated hernia through a defect of the broad ligament of uterus was in 1861 by Quain, found at autopsy [8]. Two types of hernia of the broad ligament have been classified by Hunt [9]: the fenestra type that involves a complete fenestration through a defect in the broad ligament and the pouch type that involves hernia into the pouch from an anterior or posterior aperture. The defect in our case was of the fenestra type.

**Figure 2:**
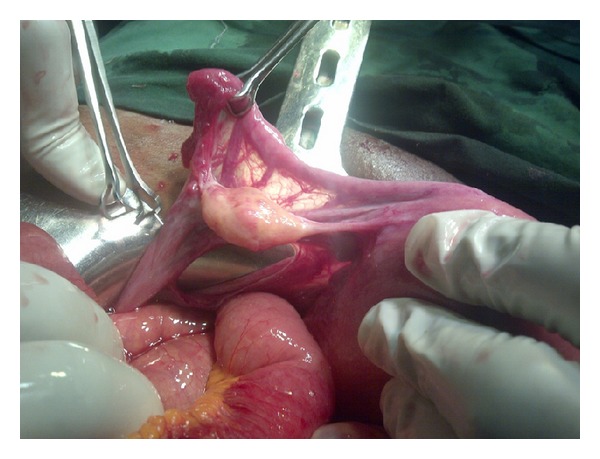
Congenital defect through the broad ligament.

**Figure 3:**
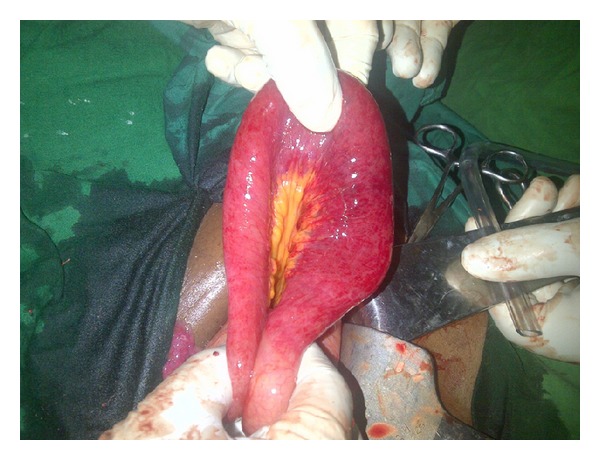
Herniated loop of small bowel.

**Figure 4:**
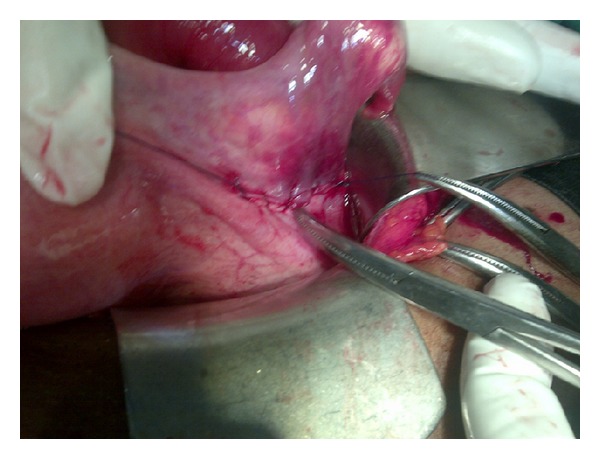
Postrepair of broad ligament defect.

Preoperative diagnosis of hernia of the broad ligament is quite difficult. The precise pathogenesis of a defect of the broad ligament remains unknown; its causes are considered to include surgery, pelvic inflammatory disease, delivery trauma, and congenital anomaly (Figure 2) [10]. Our case had neither any relevant medical history, thereby suggesting a congenital anomaly. In females, fusion of the paramesonephric ducts forms the broad ligament. The present case showed both incomplete fusion of the broad ligament and incomplete fixation of the ascending colon leading to mobile cecum. These anomalies might have resulted from some abnormalities in a similar embryonic period.

## 4. Conclusion

We emphasize that intestinal hernia through a defect of the broad ligament should be added to the list of differential diagnosis for female patients presenting with features suggestive of torsion ovarian cyst or acute abdomen without any prior history of laparotomy. Early diagnosis and surgical repair reduce morbidity and mortality from strangulation.

## References

[1] Meyers MA (1970). Paraduodenal hernias. Radiologic and arteriographic diagnosis. *Radiology*.

[2] Passas V, Karavias D, Grilias D, Birbas A (1986). Computed tomography of left paraduodenal hernia. *Journal of Computer Assisted Tomography*.

[3] Blachar A, Federle MP, Forrest Dodson S (2001). Internal herniaclinical and imaging findings in 17 patients with emphasis on CT criteria. *Radiology*.

[4] Oelsner G, Shashar D (2006). Adnexal torsion. *Clinical Obstetrics and Gynecology*.

[5] Breech LL, Hillard PJA (2005). Adnexal torsion in pediatric and adolescent girls. *Current Opinion in Obstetrics and Gynecology*.

[6] Chiou S-Y, Lev-Toaff AS, Masuda E, Feld RI, Bergin D (2007). Adnexal torsion: new clinical and imaging observations by sonography, computed tomography, and magnetic resonance imaging. *Journal of Ultrasound in Medicine*.

[7] Houry D, Abbott JT (2001). Ovarian torsion: a fifteen-year review. *Annals of Emergency Medicine*.

[8] Slezak FA, Schmeter TM, Nyhus LM, Condon RE (1995). Hernia of the broad ligament. *Hernia*.

[9] Hunt AB (1934). Fenestrate and pouches in the broad ligament as an actual and potential cause of strangulated intra-abdominal hernia. *Surgery, Gynecology & Obstetrics*.

[10] Ishihara H, Terahara M, Kigawa J, Terakawa N (1993). Strangulated herniation through a defect of the broad ligament of the uterus. *Gynecologic and Obstetric Investigation*.

